# Effects of Reciproc, ProTaper Next and WaveOne Gold on Root Canal Walls: A Stereomicroscope Analysis

**DOI:** 10.22037/iej.v13i2.16327

**Published:** 2018

**Authors:** Marcely Cassimiro, Kaline Romeiro, Luciana Gominho, Andressa de Almeida, Lívia Silva, Diana Albuquerque

**Affiliations:** a *Department of Operative Dentistry and Endodontics, Dental College of Pernambuco, University of Pernambuco (UPE), Camaragibe, PE, Brazil;*; b *Department of Odontology, Biological Sciences Unit, Federal University of Campina Grande (UFCG), Campina Grande, PB, Brazil*

**Keywords:** Dentinal Defects, Microcracks, Nickel-Titanium Instruments, Root Canal Preparation

## Abstract

**Introduction:**

The aim of this study was to analyse the potential occurrence of dentinal defects after root canal preparation using three engine-driven instruments.

**Methods and Materials:**

Eighty permanent mandibular incisors were selected. Twenty teeth did not undergo preparation, and the remaining teeth were divided into three groups (*n*=20): Reciproc (REC)*, *ProTaper Next (PTN) and WaveOne Gold (WOG). The samples were dyed with methylene blue, sectioned perpendicularly to the long axis in 3-, 6- and 9-mm slices and were finally observed under a stereomicroscope (under 25×). The absence/presence of dentinal defects was documented by two blind observers. The data were analysed using Pearson’s *chi*-squared test with a confidence level of 95% (*P*=0.05). The time to prepare the samples was recorded, and the groups were compared using *F*-test (ANOVA).

**Results:**

The control group did not present any defects, and the differences between the control and experimental groups were statistically significant (*P*<0.05). WOG, PTN and REC caused microcracks on 60%, 33.33% and 18.33% of the samples, respectively. No significant differences between the groups in the 3-mm sections (*P*=0.126) were observed. There were significant differences in the 6-mm (*P*=0.042) and 9-mm sections (*P*<0.001). When WOG and PTN were used to perform root canal preparation, a significant difference was found in the average time (*P*=0.047).

**Conclusion:**

All the used instruments caused dentinal defects in the root dentin. All the instruments were used to perform the preparation with a similar average time.

## Introduction

The chemo-mechanical root canal preparation aims to remove microorganisms, debris and tissues completely through the enlargement of the root canal diameter [1]. During the preparation, stress concentrations that originate from the contact of the endodontic instrument with the dentin may induce the formation of dentinal defects such as microcracks [2]. Through the application of repeated tension *via* occlusal forces, these dentinal microcracks may have the potential to develop into vertical root fracture (VRF) [3]. Evidence shows that VRFs are probably caused by the propagation of smaller and less pronounced dentinal defects rather than the force used during the preparation or the obturation of the root canal [4, 5]. 

Generations of nickel-titanium (NiTi) engine-driven instruments were introduced with various designs, alloy treatments and kinematics. Amongst them, the Reciproc (REC; VDW, Munich, Germany) instrument can be used to perform root canal preparation with only a single reciprocating kinematic instrument [6]. Additionally, it is built *via* heat treatment of the surface (Memory Wire; Dentsply, Tulsa Dental Specialties, Tulsa, OK, USA). The ProTaper Next system (Dentsply Maillefer, Ballaigues, Switzerland) has a similar heat treatment of the surface but with rotary kinematics. Recently, the WaveOne Gold file was released (WOG; Dentsply Maillefer, Ballaigues, Switzerland), which performs root canal preparation using a single instrument with a reciprocating movement. Moreover, it has a new heat treatment, the Gold Wire (Dentsply Maillefer, Ballaigues, Switzerland).

Multiple studies using root sectioning and analysis under microscope have shown a correlation between the preparation of the root canal using NiTi mechanical instruments and the formation of dentinal defects [3, 7-11]. In the literature, there is no consensus regarding the relationship between kinematics and different designs of NiTi instruments in the formation of dentinal damage. Thus, the present study aims to analyse the occurrence of dentinal defects after the preparation of the root canal system using different automated NiTi instruments. The null hypothesis was that there would be no significant difference in the formation of dentinal defects amongst the studied groups.

## Materials and Methods

***Sample size calculation***

The calculation of the sample size was based on the work of Liu *et al.* [6], who estimated the effect size of the dentinal defects promoted by reciprocating and rotary systems. The sum of the percentage of specimens with complete and incomplete dentinal microcracks varied from 5% to 45%. With the assistance of statistical software (Epi Info™ 6 for Windows; Centers for Disease Control and Prevention, Geórgia, EUA) and a margin of error of 5%, 15 mandibular incisors per group would be required to achieve 80% power. Thus, a sample consisting of 20 mandibular incisors yielded a power level of 92.2%.

***Sample selection***

This study was conducted with the approval of the Institutional Ethics Committee (N: 43800815.2.0000.5207). Eighty human permanent mandibular incisors with straight roots (<5^° ^curvature) [12] -that were recently extracted for therapeutic reasons from patients without parafunctional habits and periodontal problems- were selected. The curvature angles were chosen on the basis of the initial radiographs by using Image J software version 1.46r (National Institutes of Health, Bethesda, MD, USA). The teeth were disinfected in a 0.1% thymol solution for 24 h and were kept in purified filtered water until they were used. Periapical radiographs of the teeth in the buccolingual and mesiodistal directions were obtained with the intention of visualising inflammatory resorptions and calcifications as well as the presence of a single root canal. Teeth that met the exclusion criteria were replaced. All the laboratory procedures were performed by the same operator, who was a specialist in endodontics and experienced with instrumentation techniques.

**Table 1:** Buccolingual and mesiodistal dimensions of the selected teeth (mm)

**Distances/Weight**	**PTN**	**WOG**	**REC**
**Mesiodistal distance (mm)**	3.74 (0,37)	3.82 (0,44)	3.78 (0,35)
**Buccolingual distance (mm)**	5.80 (0,46)	5.83 (0,61)	5.77 (0,34)
**Weight (g)**	0.30 (0,05)	0.33 (0,07)	0.31 (0,04)

**Table 2:** Evaluation of dentinal microcracks according studied groups (%)

**Cross-sections**	**PTN N (%)**	**WOG N (%)**	**REC N (%)**	**Control N (%)**
**Total **	100 (20)	100 (20)	100 (20)	100 (20)
**3 mm**				
**Yes**	25 (5)	55 (11)	30 (6)	0
**No**	75 (15)	45 (9)	70 (14)	100 (20)
**6 mm**				
**Yes**	40 (8)	60 (12)	20 (4)	0
**No**	60 (12)	40 (8)	60 (16)	100 (20)
**9 mm**				
**Yes**	35 (7)	65 (13)	5 (1)	0
**No**	65 (13)	35 (7)	95 (19)	100 (20)

**Table 3:** Time required for root canal preparation with PTN, WOG and REC

**Time (s)**	**PTN**	**WOG**	**REC**
**Mean (SD)**	26.35 (10,01)	19.04 (8,73)	23.40 (8,61)

The coronal portions of the teeth were removed using a double-sided diamond disc with low rotation and under water refrigeration. The samples had a standard length of 13 mm. The samples were inspected under a stereomicroscope (SteREO Discovery.V12, ZEISS, Germany) with 15× magnification to detect any pre-existing cracks or fracture lines. 

All the teeth were examined and compatible with a #10 K-file made from stainless steel (Dentsply Maillefer, Ballaigues, Switzerland). The length of the canal was determined by inserting the file until the tip became visible on the apical foramen. The canal length was defined as the distance between the tip of the file and the reference plane. The working length (WL) was calculated by subtracting 1 mm from the obtained length.

To confirm that the anatomy of the teeth was similar in each group, the result of the statistical analysis revealed that the relationship between the ratio of the buccolingual dimension to the mesiodistal dimension and the average of the weights of the samples (Table 1) was not significantly different (*F*-test of ANOVA test; *P*> 0.05).

***Root canal preparation***

Prior to preparation, the periodontal ligament was simulated. Roots were immersed into molten wax and then, all the samples were embedded in acrylic resin blocks. The wax on the root surface was cleaned with the help of a curette prior to the polymerization of the acrylic resin. A silicone impression material (vinyl polysiloxane impression material, 3M ESPE, Seefeld, Germany) covered the root surface to simulate the periodontal ligament. All the roots were then embedded into acrylic resin again. 

Initially, the root canals were irrigated with 2 mL of a 2.5% sodium hypochlorite solution (NaOCl). The glide path of all the samples was made with a #10 K-file (Dentsply Maillefer, Ballaigues, Switzerland). The control group had no preparation (*n*=20). The experimental groups were prepared with the instruments, REC, PTN and WOG, according to the manufacturer recommendations. 

***Reciproc (REC)***

A single REC file (25/0.08) with reciprocating movement was used. The motor that was used was VDW Silver (VDW, Munich, Germany), which had 350 rpm and 5 N/cm^2^ of torque. The preparation was performed using in-and-out pecking movements with 3 mm of amplitude until the WL was reached with a brush motion on the buccolingual extension. 

***ProTaper Next (PTN)***

The PTN system was used in the X1 (17/0.04) and X2 (25/0.06) instrumentation sequence until the WL was reached in a continuous rotary movement. The motor that was used was the VDW Silver (VDW), which had 300 rpm and 2 N/cm^2^ of torque. In-and-out pecking movements with 3mm of amplitude were used to prepare the root canal with a brush motion on the buccolingual extension. 

***WaveOne Gold (WOG)***

The root canals were prepared with a WOG primary single file (25/0.07) in a reciprocating movement. The preparation was performed with in-and-out pecking movements of the instrument with 3 mm of amplitude until the WL was reached with a brush motion on the buccolingual extension. The used motor was the VDW Silver (VDW, Munich, Germany), which had a 350 rpm and 5 N/cm^2^ of torque. 

With the use of each instrument, the canal was irrigated with 2 mL of 2.5% NaOCl. At the end of the process, a final irrigation was performed using 2 mL of 17% EDTA and 2 mL of 2.5% NaOCl. The total volume of NaOCl that was used during the preparation was 12 mL. In the final stage, each tooth was irrigated with 5 mL of distilled water. The instruments were used only once according to the manufacturer instructions.

The total time to prepare each sample was measured in seconds with the assistance of a digital timer. This amount was obtained by calculating the average of the files usage times until they reached the WL. The time devoted to the irrigation processes, change and cleaning of the instruments was not accounted for. 

***Sectioning and microscopic examination***

The specimens were filled with 1 mL of 0.5% methylene blue solution (Sigma-Aldrich Co., Saint Louis, MO, USA) (pH=7) until the WL was reached. Then, they were immersed in dye solution and vibrated in a ultrasonic cleaner at 40 kHz for 10 min [12]. They remained immersed in dye solution for 24 h and were washed in running water and afterwards, irrigated with 5 mL of distilled water. All the samples were sectioned perpendicularly to the long axis in 3-, 6- and 9-mm slices from the root apex with a double-sided diamond disc and low rotation under water refrigeration [10]. The sections were analysed under a stereomicroscope with 25× magnification and documented to examine the presence or absence of dentinal defects [10].

**Figure 1:**
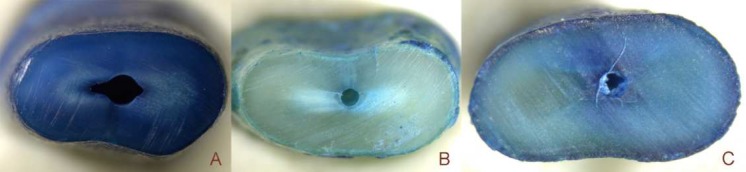
Tooth cross section showing absence/presence of microcracks; *A)* Absence of crack; *B)* After preparation; *C)* After cross sectional cut

The presence or absence of dentinal defects was classified according to the recommendations of Yoldas *et al.* [3]. The absence of defects was defined as a root dentin that did not present microcracks or any other dentinal damage (Figure 1A). The presence of defects was defined as the occurrence of any microcracks that propagated from the walls of the canal without reaching the exterior surface of dentin or that extended from the exterior surface of the dentin without reaching the lumen of the canal (Figure 1B). Fracture lines were classified as cracks that extended from the lumen of the canal to the external surface of the root. Only the microcracks that were stained by the methylene blue dye were considered (Figures 1B and 1C). For that purpose, 240 images were screened 2 times by 2 blinded and pre-calibrated endodontists. There was a 2-week interval between each analysis. When divergence occurred, the image was examined by a third observer for a final determination.

***Statistical analysis***

The results regarding the presence of dentinal defects were expressed as the number and percentage of samples with microcracks in each group, and the data was analysed using Pearson’s *Chi*-squared test. To compare the sections between the experimental groups, McNemar’s test was used. The *F*-test (ANOVA) was used to compare the groups in relation to total preparation time. The tests were performed using a confidence level of 95% (*P*=0.05). Statistical analyses were performed using SPSS (SPSS Inc., Chicago, IL, USA) version 23.

## Results

The percentages of specimens with dentinal defects in the experimental groups across all the analysed sections were: control groups (0%), REC (18.33%), PTN (33.33%) and WOG (60%). The distribution of occurrence of dentinal defects by the experimental group in each root section can be seen in Table 2. There were no cases of fracture lines in any groups; only dentinal microcracks were observed. The WOG group had the highest percentage of dentinal defects in the 3-mm (55%), 6-mm (60%) and 9-mm (65%) sections. In terms of the fewest dentinal defects, the groups that presented the lowest defect formation were the PTN group in the 3-mm (25%) section and REC group in the 6- and 9-mm sections (20% and 5%, respectively). Given a fixed margin of error (5%), a significant difference was demonstrated between the groups (*P*<0.05) in each of the sections. Considering only the experimental groups, there was no significant difference between the groups in the 3-mm (*P*=0.126) sections. However, a significant difference existed in the 6-mm (*P*=0.042) and 9-mm (*P*<0.001) sections. 

The average time needed to prepare the root canals using each of the evaluated instruments can be observed in Table 3. The WOG and PTN groups were significantly different in terms of the average preparation time of the root canal (*P*=0.047).

## Discussion

All experimental groups had dentinal defects, corroborating the findings of several studies [2, 6, 13, 14]. In addition, there was a significant difference across the groups. Therefore, the null hypothesis was rejected since there would be no difference in the formation of defects among the studied instruments. 

Kim *et al.* [15] described the potential correlation between the design of NiTi rotary instruments and the formation of dentinal defects. They observed that the high stress concentration in the walls of the root canal system caused by those instruments increases the risk of dentinal damage that is created. According to Yoldas *et al*. [3], the formation of dentinal microcracks could be related to the design of the tip of rotary instruments, the geometry of the cross-section, the taper type (constant or gradual), constant or variable step and finally the form of the cannelure. In the present study, dentinal defects occurred independently of the kind of the used instruments, sequence of rotary instruments or reciprocating single files. However, the experimental preparation groups varied in their design, cross-section, tip design and taper. They were similar only in the size (#25) of the tip. 

It is speculated that another aspect which reduces the production of tension on dentinal walls is the flexibility of the instrument that is provided by the heat treatment of the NiTi alloys. However, the flexibility can be influenced by the design of the instrument [16]. Consistent with this idea, the results of the present study showed that the largest number of dentinal defects was promoted by the WOG instrument, which has a high level of flexibility due to its heat treatment of NiTi alloy and its parallelogram-shaped cross-section [17].

The design of the cross-section of the instrument can influence the number of times that it touches the root dentin, creating the potential to provoke different degrees of tension. More contact of the instrument with the walls of the canal can induce the formation of dentinal defects [2]. The REC instrument has an S-shaped cross-section with two cutting edges and a positive angle, which provides excellent efficiency in the removal of the dentin [18, 19]. The PTN system has an off-centred rectangular cross-section [9] with two cutting edges and eccentric movement, which minimizes the contact with the dentin [20]. The WOG single file has a cross-section that alternates touches on the dentin with 2 and 1 edges during a 360^°^ rotation. In this way, the contact of this instrument with the dentinal walls might increase, promoting the formation of dentinal defects, which supports our findings. 

However, Versluis *et al.* [21] concluded that during canal preparation, there is a high concentration of tension on the buccolingual extensions in addition to the medium and cervical thirds. This contention was verified in the present study, as the WOG instrument had the highest percentage of touches on the dentin in 6- and 9-mm sections. 

The initial tapers of the studied instruments were different. The REC file had a tip with a 0.08 taper, whereas the WOG file had an initial taper of 0.07 and the PTN file had a 0.06 taper. It is possible that the taper of the tip is not a critical factor in the formation of dentinal defects at the apical level, as there was no significant difference among the instruments 3 mm from the apex. 

During the preparation of the canal with NiTi rotary systems, a varying degree of rotary force is applied to the root canal walls with the potential to produce dentinal defects [22]. Reciprocating movement could prevent the continuous rotary force and constant torque that are applied to the walls of the canal [23], resulting in less damage than rotary movement. However, in the present study, the reciprocating files, REC and WOG, alternated between the lowest and highest frequency of microcracks, respectively. Thus, kinematics is not related to the formation of dentinal defects. 

The WOG and REC instruments that were used to perform the preparation of the canal had a similar average preparation time, without any significant difference among them. However, the PTN group presented a significant difference in comparison with the WOG group that was probably caused by the use of two instruments on the WL.

Mandibular incisors with a single canal were selected to minimize the effect of the variation in anatomical complexity. The canals of these teeth had small anatomical diameters in the apex[10], making them compatible with the preparation size 25. The simulation of periodontal ligaments was performed in several studies [9, 10, 13, 23]. It serves primarily to absorb the tension associated with the preparation [24], therefore, the analysis of the effects of the instruments can be more trustworthy. 

The use of methylene blue dye after the preparation of the root canals was crucial to the differentiation of the defects caused by the instrumentation and sectioning of the sample. The unstained dentinal defects were not accounted for, as any defect which did not get in contact with the dye after the sectioning. The presence of dentinal defects was not observed in the control group after sectioning of the samples. These findings are consistent with the results of several studies [3, 8, 10]. 

Currently, several methods are employed to evaluate the formation of dentinal defects. The use of extracted teeth with observation under stereomicroscope or scanning electron microscope (SEM) after sectioning of the samples is one of the most common methods [2, 11, 14, 25]. The use of human and animal mandibles to conserve the periodontal ligaments, *in situ* preparation and analysis of dentinal defects under microscopy have been proposed. However, the results are conflicting, as they do not indicate the formation of dentinal defects after the preparation [26] or the impossibility of comparing the control group and the experimental groups due to the presence of defects in both. Recently, the computed tomography technique, which does not damage the sample and provides high accuracy, has been used. However, it yields divergent results of the evaluation of dentinal defects. Researchers agree that there are pre-existing dentinal defects [27-29]; however, they have not reached consensus concerning posterior formation [27-29]. 

It is still not clear if dentinal defects can become fractures after the preparation of the root canal, as even teeth without any endodontic treatment can still develop fracture [7]. Therefore, at this time, there is no definitive conclusion concerning the clinical implications of these dentinal defects in long term [30]. More studies on this topic and the development of more effective methods and analysis are necessary.

## Conclusion

It can be concluded that REC, WOG, and PTN instruments can cause formation of dentinal defects in mandibular incisors. The REC file generated the lowest incidence of defects, and the highest incidence occurred 6 and 9 mm from the root apex. All the instruments that were used to perform the preparation of the root canal had similar preparation times.
